# *Quo natas, Danio*?*—*Recent Progress in Modeling Cancer in Zebrafish

**DOI:** 10.3389/fonc.2017.00186

**Published:** 2017-08-28

**Authors:** Stefanie Kirchberger, Caterina Sturtzel, Susana Pascoal, Martin Distel

**Affiliations:** ^1^St. Anna Kinderkrebsforschung, Children’s Cancer Research Institute, Innovative Cancer Models, Vienna, Austria

**Keywords:** zebrafish, cancer, xenograft models, genetically engineered models, tumor microenvironment, compound screen

## Abstract

Over the last decade, zebrafish has proven to be a powerful model in cancer research. Zebrafish form tumors that histologically and genetically resemble human cancers. The live imaging and cost-effective compound screening possible with zebrafish especially complement classic mouse cancer models. Here, we report recent progress in the field, including genetically engineered zebrafish cancer models, xenotransplantation of human cancer cells into zebrafish, promising approaches toward live investigation of the tumor microenvironment, and identification of therapeutic strategies by performing compound screens on zebrafish cancer models. Given the recent advances in genome editing, personalized zebrafish cancer models are now a realistic possibility. In addition, ongoing automation will soon allow high-throughput compound screening using zebrafish cancer models to be part of preclinical precision medicine approaches.

## Zebrafish as a Model Organism in Cancer Research

George Streisinger established zebrafish, a small freshwater fish naturally found in rice fields and tributaries to the river Ganges, as a vertebrate model organism in his 1981 Nature publication “Production of clones of homozygous diploid zebra fish (*Brachydanio rerio*)” (1, 2). Since then, supported by large mutagenesis screens, zebrafish has become one of the major model organisms in vertebrate genetics and developmental biology (3, 4). Roughly two decades later, the potential of the zebrafish model to study human diseases began to be exploited [reviewed in Ref. (5)]. Especially, characteristics like the fast development outside the mother, transparency at embryonic and larval stages, and the high number of offspring allowing for live imaging and cost-effective compound screening make the zebrafish model an attractive complementary model to more classical mouse models.

Disease modeling in zebrafish was boosted further when the zebrafish reference genome, published in 2013, revealed that zebrafish possess >80% of all human disease-related genes, indicating that many human diseases can, in fact, be modeled in zebrafish (6). This also includes cancer and in the early 2000s, pioneering transgenic models for leukemia and rhabdomyosarcoma were established by the Crosier, Look, and Zon laboratories (7–9). From *Xiphophorus* melanoma models, it was already known for decades that fish can serve as a useful model to investigate tumor driving mechanisms [reviewed in Ref. (10)]. However, cancer research in zebrafish particularly benefits from the many genetic tools and transgenic strains established by the zebrafish community over the years. For many cell types, e.g., hematopoietic cells, a specific transgenic strain is readily available demarcating distinct cell types like neutrophils, macrophages, B cells or T cells and natural killer cells by fluorescent protein expression (11–14). Availability of such transgenic strains offers a direct readout for effects of oncogenes on distinct cell populations by confocal microscopy and also quantification by flow cytometry. In addition, cellular interactions of labeled cells, e.g., within the tumor microenvironment (TME), can be directly monitored. Furthermore, targeted oncogene expression can be achieved using gene expression systems like Gal4/*UAS* or Cre/*loxP*. Through enhancer and gene trap screens, many cell type-specific Gal4 and Cre zebrafish strains have been established and await their application in cancer research (15–19).

## Modeling Approaches in Zebrafish

**Figure 1:**
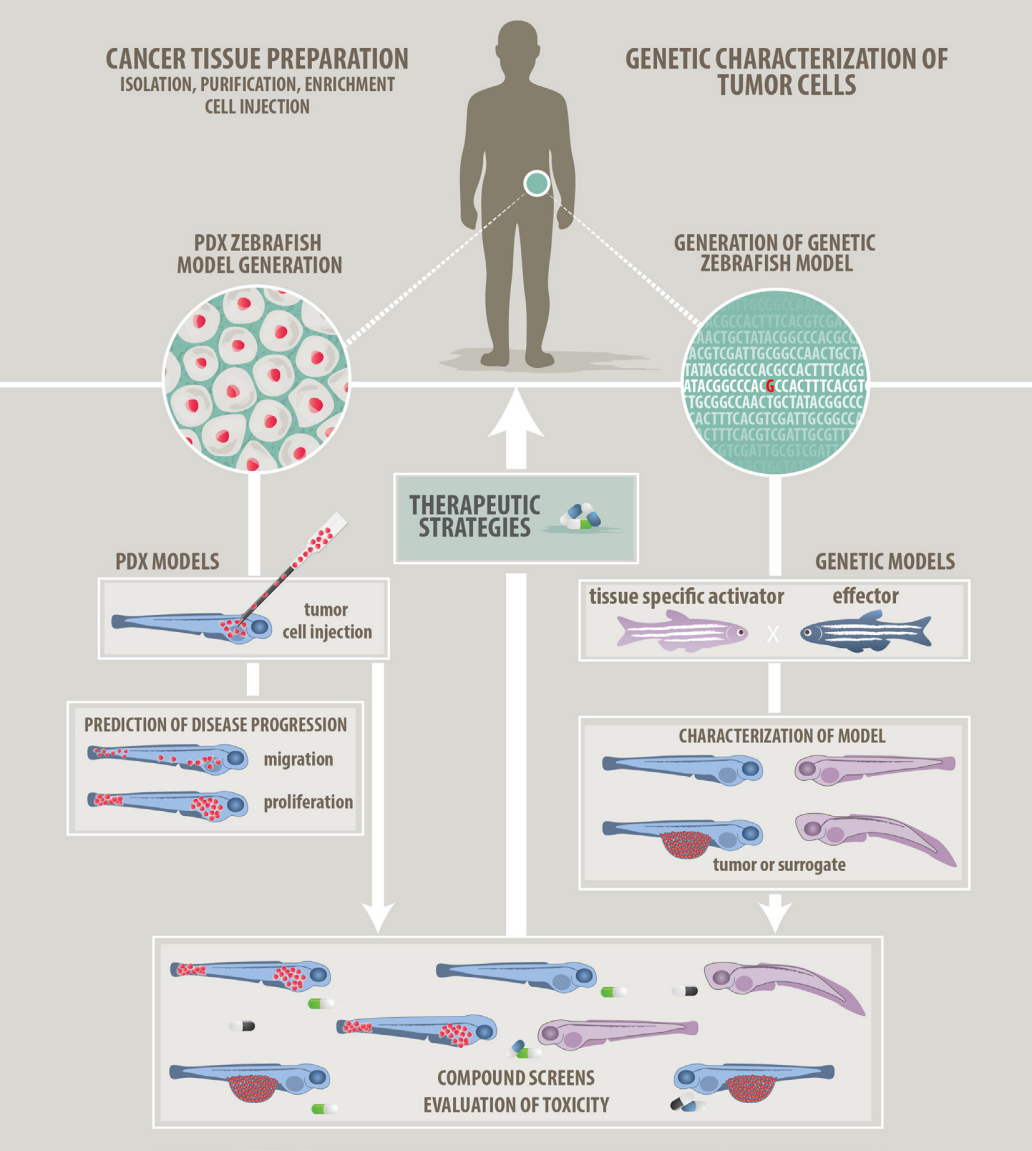
Approaches to modeling cancer in zebrafish. We describe two main approaches how zebrafish can be used in cancer research and how zebrafish will help to develop patient-tailored therapies in the future. (*Left panel*) *Patient-derived xenograft approach*: cancer cells prepared from resected or isolated patient material will be transplanted into zebrafish larvae. Monitoring of *in vivo* proliferation, migratory behavior, and interaction with host cells like endothelial cells might allow predictions of aggressiveness and disease progression. (*Right panel*) *Genetic modeling approach*: bioinformatic analyzes of Omics data will point at candidate target genes. Genetic models featuring single or combined mutations will be generated using the zebrafish tool kit. Genetic models will be used for *in vivo* investigation of tumorigenesis. In addition, a screenable phenotype will be identified. This can be an actual tumor, hyperproliferating cells, or developmental abnormalities. Studies of the tumor microenvironment are also possible on genetic models. (*Common middle panel*) *Compound evaluation, compound screens, and development of therapeutic strategies*: testing of single compounds, compound synergies, evaluation of toxicity, and screening for new compounds will help to advise on optimized and in the future individualized therapies.

In this review, we will focus on two fundamentally different cancer modeling approaches being pursued in zebrafish at the moment: genetic and xenograft approaches (Figure 1). In addition, syngeneic and allogeneic cell transplantation using genetic zebrafish models has given insight into evolution and heterogeneity of cancer cells and their tumor-propagating and self-renewal potential (20, 21). This strategy has been reviewed in detail recently by Moore and Langenau (22).

Genetic approaches are based on the transfer of mutations found in cancer cells from the patient to zebrafish to investigate functional consequences of the respective mutation. This is achieved not only by expressing a mutated human gene in zebrafish but also by mutating the orthologous zebrafish gene or even by expressing cancer-related genes from other species in zebrafish like mouse *c-myc* or *xmrk* from *Xiphophorus* (8, 23). Available genetic tools and strategies for expression of oncogenes and emerging technologies to study tumor suppressors are discussed below. With next-generation sequencing (NGS) revealing the mutational landscape of many tumor genomes, new challenges have arisen. How are the many mutations best evaluated functionally if they constitute driver, modifier, or passenger mutations? Here, the zebrafish model has the potential to offer solutions through functional testing of single-mutated genes and mutation combinations.

Genetically engineered zebrafish models (GEZMs) allow for characterization of cell autonomous and non-cell autonomous mechanisms driving tumorigenesis within an intact organism. Such insights will instruct the development of therapeutic strategies. Ideally, genetic zebrafish cancer models present with an early phenotype, so that they can be used in compound screens on embryos or larvae to identify compounds able to eradicate tumor cells. Due to these obvious advantages, genetic cancer modeling in zebrafish is rapidly growing, and we will report on recent progress and discuss what still needs to be done.

Xenografting of patient-derived cancer cells into zebrafish promises to be an alternative to current patient-derived xenograft (PDX) models in mouse. In particular, transplantations into zebrafish embryos and larvae appear appealing as tumor cells can be observed directly in the transparent host and their proliferation and migratory behavior can be monitored by live microscopy. By this means, the interaction of the tumor cells with the host environment, including biological processes like neovascularization, can also be investigated. Probably most important, zebrafish larvae are ideal for higher throughput screens to identify compounds able to eradicate or differentiate tumor cells. Of particular interest is that such short-term zebrafish PDX models typically provide insights in less than 2 weeks and thus could potentially provide information relevant to patient treatment. For example, the model could assess the aggressiveness of a tumor, thereby helping estimate disease progression, or could be used to develop therapeutic strategies, based on *in vivo* compound evaluation or a compound screen, within a time frame relevant to the respective patient. However, PDX models in zebrafish (PDXz) are still in their infancy, robust PDX protocols are still missing, and several obstacles need to be overcome in reaching this rewarding aim. We will report on the progress and the challenges in the zebrafish xenograft field below.

## The Tool Kit for Genetic Zebrafish Cancer Models

Genetic zebrafish cancer models are often based on cell type-specific expression of human oncogenes to induce tumors mimicking the related human tumor entity. For this, typical promoter-oncogene constructs as well as inducible (e.g., heatshock) and bipartite expression systems like Gal4/*UAS*, Cre/*loxP*, and lexA/*lexAOP* are used. An advantage of the bipartite and inducible gene expression systems, and combinations of the two (e.g., Tet-ON, Cre^ERT2^/*loxP*), is their ability to circumvent oncogene-related lethality prior to sexual maturity, which interferes with creation of transgenic strains. One example of an effective zebrafish cancer model was created by driving *KRAS^G12V^* specifically in the liver using the inducible Cre^ERT2^ system. The resulting fish developed various liver tumors ranging from benign adenoma to malignant hepatocellular-carcinoma and -blastoma (24). Furthermore, inducible conditional systems have been successfully used to study oncogene addiction. In a mifepristone-inducible model of zebrafish myca/b overexpression, it was shown that liver carcinogenesis was reversible upon withdrawal of the drug (25). Interestingly, regression was even independent of p53 as it also occurred in the *p53* mutant background.

While the introduction of dominant oncogenes is straightforward, studying tumor suppressors has been more difficult and initially relied on identifying fish created through random mutagenesis screens using chemicals such as ethylnitrosourea (ENU) (26, 27) or by insertional mutagenesis (28). Zinc-finger nucleases and transcription activator-like effector nucleases (TALENs) provided the first means of creating targeted knockout animals and were quickly adopted for the generation of cancer models. For example, neurofibromatosis 1 (*nf1a* and *nf1b*) zebrafish mutants were successfully created with zinc-finger nucleases (29) and tumor-suppressor retinoblastoma 1 mutants with TALENs (30).

While TALEN and zinc-finger nuclease-based methods do produce mutations, they are inefficient and labor-intensive. However, the recent advent of highly effective CRISPR/Cas9 technology provides unprecedented possibilities for genome editing in zebrafish, including for the creation of cancer models. Custom-made CRISPR guide RNAs facilitate rapid screens for tumor suppressors or cancer modifiers. Frequent bi-allelic targeting observed with CRISPR/Cas9 saves time spent back-crossing fish lines to homozygosity. Cell type-specific knockouts can be achieved by expression of Cas9 under a tissue-specific promoter allowing for spatial control of gene disruption in somatic cells (31). Although still a challenge, progress has been made in establishing knockin strategies targeting an endogenous cancer-relevant locus by homologous recombination (32–34). In the future, this will facilitate the generation of conditional knockout lines by introducing flanking *loxP* sites into tumor suppressors. Crossing such lines with tissue-specific inducible Cre lines (e.g., tamoxifen-inducible Cre^ERT2^) will provide temporal and spatial control over the gene-disrupting event to generate driver or modifier mutations in zebrafish cancer models. In the future, even personalized CRISPR/Cas9 genetically engineered zebrafish cancer models appear feasible. A comprehensive overview focusing on genetic tools and their application in conditional zebrafish cancer models was also recently published by Mayrhofer and Mione (35).

## Progress in Genetic Cancer Modeling in Zebrafish

In the past, the zebrafish cancer field was dominated by genetic models for only a few types of cancer: melanoma (36, 37), neuroblastoma (38), rhabdomyosarcoma (9), leukemia (specifically T-ALL) (8), and liver cancer (39, 40) [reviewed in Ref. (41–44)].

Recently, researchers have created several promising new zebrafish models and improved existing ones to better address specific questions (Table 1 lists recent models according to tumor entity). In the following, we will highlight several recent examples.

**Table 1:** Recently developed and improved zebrafish cancer models.

Cancer entity	Tissue driver: oncogene	Tumor suppressor	Modifier	Tumor frequency/survival	Effective compounds	Reference
Neuroblastoma	*dbh:MYCN*		*ALK^F117L^*	5% neuroblastoma at 24 wpf		Zhu et al. (45)

	*dbh:MYCN*	*nf1a^−/−^, nf1b^+/−^*		*nf1a^−/−^*: 60% neuroblastoma at 4 wpf; *nfla^−/−^ nf1b^+/−^*: 82% neuroblastoma	Trametinib, isoretinoin	He et al. (46)

Malignant peripheral nerve sheath tumor (MPNST)	*ia2:EGFP* (15 Mb deletion of chromosome 1)			30% MPNST at 30 mpf		Astone et al. (47)

	*Sox10:PDGFRA^wt or mut^*	tp53^−/−^, nfla*’’, nf1b^−/−^		*PDGFRA^wt^*: 80% at 30 wpf, *PDGFRA^mut^*: 50% at 30 wpf	Sunitinib, trametinib	Ki et al. (48)

		*tp53^−/−^*	*atg5^K130R^*	*p53^+/−^*: 15% tumors vs. *p53^+/−^ mitfa:atg5 ^KI30R^*: 40% tumors		Lee et al. (49)

Brain cancer		*rb1*		*33%* tumors in fish at 18 mpf injected with exon 2 or 3 transcription activator-like effector nuclease		Solin et al. (30)

	*krt5:KRAS^G12V^ or gfap:KRAS^G12V^*			*krt5:KRAS^G12V^* 26% at 1 mpf, 50% at 12 mpf; *gfap:KRAS^G12V^: 50%* brain tumors at 12 mpf		Ju et al. (50)

	*zic4:Gal4 inj. uas:HRAS^G12V^*		*YAP*	*YAP^SSA^* (dominant-active) reduces survival from 60 to 4% in *zic4:HRAS^G12V^*		Mayrhofer et al. (51)

	*sox10:NRAS* or *NRAS^Q61R^*	*tp53^−/−^*		50% CNS-PNET tumors at 6 wpf	MEK inhibitor AZD6244	Modzelewska et al. (52)

Eye cancer	*krt5:Ga14; 14xuas; zfSmoa1*			80% optical pathway glioma and retinal tumors at 12 mpf		Ju et al. (53)

Leukemia	*spi1:lox-NUP98-HOX9 × hsp70:Cre*		*meis1, Cox2*	25% myeloproliferative neoplasms between 19 and 23 mpf	COX and HDAC inhibitors	Deveau et al. (54)

	*c-myb^hyper^* (gene duplication, wt, and truncated gene version)			Myelodysplastic syndrom, 2% progress to AML or ALL, respectively, at 10–24 mpf	Flavopiridol	Liu et al. (55)

Myeloproliferative disease		*c-cbl^−/−^*		*c-cbl^−/−^* lethal before 15 dpf, myeloid/erythroid lineages increased		Peng et al. (56)

Mastocytosis	*actb2:KIT^D816V^*			50% prevalance, 15 mpf median age of onset		Balci et al. (57)

Melanoma	*mitfa:BRAF^V600E^*	*tp53^−/−^*	*EDN3*	Cell line transplantations		Kim et al. (58)

	*mitfa:HRAS^G12v^*			30% increase of melanocytes 5 dpf	MEK inhibitor PD185342 and rapamycin	Fernandez Del Ama et al. (59)

Uveal melanoma	*mitfa:GNAQ^Q209P^*			33% uveal tumors at 5 mpf		Mouti et al. (60)

Thyroid cancer	*tg:BRAF^V600E^*			64% invasive thyroid cancer at 12 mpf		Anelli et al. (37)

Liver cancer	*fabp10:LexPR × LexA OP:myca or LexA:mycb*			Cellular alterations from 10 days post mifepristone induction (dpi), 5% hepatocellular carcinoma (HCC) at 8 mpi		Sun et al. (25)

	*fabp10:pt-beta-Catenin*			4–5 mpf enlarged livers, HCC histology, decreased survival rate	JNK inhibitors and anti-depressants	Evason et al. (61)

	*fabp10:LexPR × LexA OP:Cre × fabp10:loxp-Stop-loxp-KRAS^G12V^*			Induced with mifepristone at 4 wpf for 36 h ca. 60% tumor penetrance at 24 wpi		Nguyen et al. (24)

*dpf: days post fertilization; wpf: weeks post fertilization; mpf: months post fertilization; dpi: days post induction*.

One of the key questions is how well zebrafish models can portray human cancer, and recent data in fact revealed striking similarities between zebrafish and human cancers. In one study, the molecular resemblance between human hepatocellular carcinomas (HCCs) and zebrafish liver cancer models was analyzed (62). All of the zebrafish models use the liver-specific promoter *fabp10* to drive one of the oncogenes *myc, KRAS^G12V^*, or *xmrk* (23, 40, 63). Comparative transcriptome analysis using RNA-seq revealed that these three models together represented gene signatures of almost half (47%) of human HCC. They identified a conserved molecular pattern of 21 upregulated and 16 downregulated genes, which was not only common to the three zebrafish models but also consistent with human HCCs. This indicates that subtypes of human HCC are well represented by zebrafish models. It also shows the need for new models targeting the molecular mechanisms so far not covered by the existing mutations.

Primitive neuroectodermal tumors of the central nervous system (CNS-PNETs) are poorly understood, aggressive pediatric brain tumors with poor prognoses. Recently, a novel zebrafish tumor model for CNS-PNET was generated by expressing human wild-type *NRAS* or *NRAS^Q61R^* under the *sox10* promoter (52). The fish develop tumors in the optic tectum, cerebellum, and brain stem, and the tumors in the anterior lobes histologically and genetically resemble CNS-PNETs, specifically oligoneural and NB-*FOXR2* CNS-PNETs. In an elegant transplantation assay, the authors also showed that CNS-PNETs are sensitive to MEK inhibition.

Another new brain tumor model addresses the question of why a particular founding mutation will lead to brain lesions that are in some cases benign and in others malignant (51). The model was generated by driving *EGFP-HRAS^G12V^* expression in the central nervous system using the *zic4:KalTA4* activator strain (17), and somatic mosaic expression led to tumors mostly in the telencephalon. Interestingly, malformations with and without GFP expression could appear even in the same brains, the former an infiltrative cancer with persistent pERK activation, and the latter a sharply circumscribed heterotopia with no pERK. Comparing the tumor transcriptome to 840 human GBM markers (64) revealed that the zebrafish tumors resemble the human mesenchymal GBM subtype. Within the upregulated genes were five genes related to YAP signaling. Applying an eight gene signature featuring YAP targets to human tumors established that YAP can distinguish between mesenchymal glioblastoma and low-grade glioma and therefore could prove useful as molecular diagnostic tool. In support of the importance of Hippo signaling to tumor behavior, coexpression of a dominant-active form of YAP (YAP^S5a^) with *HRAS^G12V^* in this model led to a shift from benign heterotopias to malignant lesions with increased proliferation and reduced survival.

These examples demonstrate not only the histological but also genetic resemblance of zebrafish cancer models to their human counterparts. Importantly, the zebrafish models have direct clinical implications for human patients—they can be used to develop valuable diagnostic markers that discriminate between benign and malignant tumors, and to test possible treatment strategies. Furthermore, zebrafish models are ideal for functionally characterizing candidate variant genes and for studying the synergy of mutations found in human tumors *in vivo*, as we will discuss in the next paragraph.

### Functional Investigation of Mutations and of Alterations in Pathway Activity

In recent precision medicine approaches, NGS is increasingly used to evaluate tumors for mutations that may indicate potential treatment targets or may constitute risk factors like a high chance of metastasis. In addition, gene expression analysis of tumor cells reveals alterations in signaling pathway activity. Such knowledge is important for patient stratification to ideally provide individually tailored treatments. However, to understand the significance of the identified mutations, combinations of mutations and changes in activity of signaling pathways, the abnormalities need to be tested in a functional assay. Zebrafish is an ideal vertebrate model for *in vivo* analysis of such alterations for many reasons, including ease of genetic manipulation, accessibility from the one-cell stage, rapid development, and transparency of the embryos. Two recent examples of functionally testing mutations and signaling pathway alterations in zebrafish were in neuroblastoma and malignant peripheral nerve sheath tumor (MPNST) models, each revealing synergism between tested alterations.

Neuroblastoma, which affects the peripheral sympathetic nervous system, is one of the most frequent childhood cancers (8–10% of all childhood cancers). While the original zebrafish neuroblastoma models were based on the overexpression of human MYCN, a recent variation combined MYCN overexpression under the dopamine-β-hydroxylase (*dβh*) promoter with expression of mutated human ALK (45). The result was a dramatic increase in frequency of adrenal neuroblastoma, from 15 to 55%, caused by the combination of MYCN preventing differentiation of neuroblasts into chromaffin cells and ALK providing survival signals. More recently, the role of *nf1* mutations, which are associated with a poor outcome in human neuroblastoma, was also analyzed in the zebrafish *MYCN* model (46). An *nf1* mutation increased the tumor penetrance in MYCN-overexpressing fish to over 80% by blocking the apoptosis normally seen in those fish. The loss of NF1 led to aberrant Ras–Mapk pathway activation that can be rescued by expression of the GTPase-activating protein-related domain (GRD) of NF1. The authors further used their zebrafish model to develop a treatment strategy. By targeting the Ras/Mapk pathway with the FDA-approved MEK inhibitor trametinib in conjunction with the use of the neuroblastoma drug isotretinoin, they worked out the ideal synergistic dosage combination for maximum effect on tumor growth.

Malignant peripheral nerve sheath tumors are very aggressive soft tissue sarcomas, thought to originate from neural crest cells. About half of them arise in children with neurofibromatosis type 1, an inherited genetic disease caused by mutations in *NF1*. Prognosis is rather poor and the recurrence rate is high. So far, the therapeutic possibilities are very limited, and chemotherapy is often ineffective, leaving complete surgical resection as the best option. In recent years, a number of zebrafish models have been developed to study the molecular mechanisms underlying the disease, as well as to screen for alternative treatment options. The first model in zebrafish was based on a mutation in the tumor-suppressor *p53* leading to MPNST in around 30% of fish after 16 months (65). The long latency in patients as well as in the zebrafish model indicated that additional mutations are needed for MPNSTs to develop. PDGFRA is found to be expressed at high levels in MPNSTs. Overexpressing either wild-type or mutant PDGFRA in *p53^M214K^ nf1b^−/−^* zebrafish accelerated tumor development (48). Interestingly, overexpression of wild-type PDGFRA was even more detrimental than an activating mutation in PDGFRA leading to a tumor incidence of 80 vs. 50% at 30 weeks. This surprising reduction in tumor growth by constitutively active PDGFRA can be explained by the induction of senescence through a supra-optimal Erk and Akt downstream signal. In line with these observations, *PDGFRA* is rarely mutated in clinical samples. Using the RTK inhibitor, sunitinib together with the MEK inhibitor trametinib could efficiently inhibit tumor growth in this model.

Autophagy is a pathway involved in cellular degradation in response to starvation and cellular stress, but its role in tumorigenesis is controversial. A zebrafish MPNST model was recently used to analyze autophagy in tumor development (49). On a *p53* heterozygous mutant background, autophagy was inhibited by expressing dominant-negative *atg5^K130R^* under the *mitfa* promoter, directing expression to neural crest cells and melanocytes. Inhibition of autophagy accelerated tumorigenesis, leading mainly to MPNST and to a lesser extent to neuroendocrine and small round cell tumors. Surprisingly, given the use of the mitfa promoter, the fish did not develop melanomas. In this model, autophagy is suspected to promote genomic stability by delaying *p53* loss of heterozygosity. Inhibition of autophagy is not oncogenic by itself in this model but modulates preexisting cancer susceptibility. This shows that zebrafish models are well suited to study the contribution of cellular processes such as autophagy to cancer *in vivo* and can add an alternative perspective on data gained from mouse models and human cell lines.

### Unraveling Mechanisms of Drug Resistance by Cross-Species Oncogenomics

So far, we have presented examples demonstrating the histological and genetic similarities between zebrafish and human cancers. We have also covered how mutations can be functionally analyzed, and how synergy can be studied in zebrafish cancer models. Most models were also used to develop therapeutic strategies, which might translate to the clinic. However, targeted therapies often lead to development of resistance, and the field is in dire need for a better understanding of the underlying mechanisms of drug resistance. In the following paragraph, we highlight a recent study suggesting that drug resistance mechanisms are conserved between zebrafish and human and thus can be studied in zebrafish models.

To understand the genetic alterations underlying progression of melanoma and the development of drug resistance, an elegant cross-species oncogenomics approach was applied using a zebrafish melanoma model (66). *BRAF^V600E^*-, *NRAS^Q31K^*-, and *HRAS^G12V^*-mediated zebrafish models exist (36, 67, 68). The melanoma model driven by human *BRAF^V600E^* and mutant *p53* shows a latency of 4–6 months until melanoma manifests, indicating that additional mutations need to be acquired. Indeed, sequencing a melanoma cell line [ZMEL1 (69)] derived from this model revealed >3,000 new mutations in malignant cells. Additional treatment of ZMEL1 cells with the BRAF inhibitor vemurafenib for 4 months led to development of drug resistance. Gene expression profiling of the resistant cells (ZMEL1R) showed altered cAMP and PKA signaling, highly similar to human drug resistant samples. On the genomic level, only three additional mutations were found in drug resistant ZMEL1R cells in *bub1ba, col16a1*, and *pink1*. Strikingly, an increased mutation frequency in these genes is also observed in patient samples, suggesting that core drug resistance mechanisms are conserved between human and zebrafish. Zebrafish cancer models can thus be used to efficiently filter human sequencing data. Mutations conserved across species might offer new therapeutic strategies to overcome drug resistance.

### Visualizing Reactivation of Developmental Programs in Melanoma Formation

Studies on melanoma in zebrafish have now advanced from establishing relevant models to a stage where new insights on the regulation of tumor initiation and cellular plasticity can be gained. A concept in the cancer field is that developmental programs are reactivated during tumorigenesis and can have important effects such as regained self-renewal capabilities and migratory behavior leading to proliferation, invasion, and metastasis (70). One advantage of zebrafish here is the ability to image the cells *in vivo* and over time. Indeed, combining a zebrafish melanoma model with a reporter for *crestin* revealed that cells reverted to an embryonic neural crest state (71). *Crestin* is a gene normally only expressed during the embryonic period in neural crest cells but is also commonly re-expressed in melanoma. Using this fluorescent *crestin* reporter, the authors could follow single melanocytes in a “cancerized field” starting to express *crestin* with these clones developing into melanoma. The functional relevance of reactivating neural crest identity was demonstrated in an experiment showing that overexpression of the neural crest regulator Sox10 in melanocytes accelerated melanoma formation. Interestingly, super-enhancers regulate the neural crest progenitor signature. This is also the case for zebrafish melanomas and human melanoma lines, which share super-enhancer signatures for the neural crest transcription factors Sox10 and Dlx2. These mechanistic insights into the regulation of the embryonic neural crest program in melanoma could be exploited to develop new therapeutic strategies directed at the re-emergence of the neural crest signature, e.g., by targeting epigenetic mechanisms. In addition, key transcriptional regulators of reactivated developmental programs could potentially be used as biomarkers for early detection of oncogenesis.

## TME Studies Using Genetic Zebrafish Models

Several aspects of tumor initiation, progression, and metastasis are intimately linked with the TME. For example, induction of angiogenesis by tumor cells in a process termed “angiogenic switch” is one of the hallmarks of cancer, and neovascularization is necessary for tumor growth (72). The TME is ideally studied *in vivo* due to its complex composition of multiple cell types, including but not limited to tumor cells, immune cells, fibroblasts, and endothelial cells. In pioneering studies, Feng and Martin showed that zebrafish is a suitable model organism to study interactions between oncogene-expressing cells and innate immune cells. Using a zebrafish melanoma model, they found that *HRAS^G12V^*-expressing melanoblasts and goblet cells attract leukocytes by secreting H_2_O_2_ (73). In addition, macrophages and neutrophils provided trophic factors like prostaglandin E2, fueling proliferation of *HRAS^G12V^* + cells at tumor-initiating stages (74) [and summarized in Ref. (75)]. These findings reveal that, like in humans, pro-tumor immune cells also exist in zebrafish. We will focus on the latest progress in the field of TME studies in zebrafish in the following section.

In virally caused HCC, chronic inflammation is an important etiological factor and generally inflammation has been recognized as a hallmark of cancer (72). In a zebrafish *KRAS^G12V^* liver cancer model, neutrophils were found to be recruited to the liver (76) similar to the recruitment seen in human cancers of the digestive tract (77, 78). In this zebrafish model, neutrophils contributed to tumor growth as inhibition of neutrophil NADPH oxidase and blocking of neutrophil differentiation by a *gcsfr* morpholino led to a reduction in liver size and histological improvement. Neutrophils in the *KRAS^G12V^* + livers behaved more stationary within the tumor and morphological analysis revealed an increase in neutrophil numbers with hyper-segmented nuclei in the TME. Also, the TME was modulated by hepatocyte-produced TGF-β. As in mouse models, TGF-β induced a pro-tumor neutrophil cytokine expression pattern in zebrafish in this study, showing that essential mechanisms in the TME are conserved. Once the neutrophil-derived factors promoting liver carcinogenesis have been identified in this model, it will be interesting to see their role in human carcinogenesis.

The same group also found a possible explanation for the gender disparity in HCC, with men being more likely to develop HCC and also showing more aggressive disease progression than women. In their inducible *KRAS^G12V^* HCC model, they found increased numbers of tumor-associated neutrophils (TANs) and macrophages (TAMs) in male zebrafish, which also showed accelerated liver carcinogenesis compared to their female counterparts (79). The authors showed that male zebrafish had higher levels of cortisol, which induced expression of TGF-β. TGF-β1 in turn served as chemoattractant recruiting TANs and TAMs. Strikingly, higher cortisol and TGF-β1 levels together with higher TAN/TAM infiltration were also observed in human HCC patients indicating a causative link to tumor aggressiveness. The authors also emphasized that zebrafish is an ideal model to study cortisol-elicited effects, “as both human and zebrafish utilize cortisol as their main stress hormone whereas mouse and rat make use of corticosterone instead” (79).

Zebrafish is also a suitable model to study neo-angiogenesis as core mechanisms are conserved. Using a transgenic hypoxia reporter *Tg(phd3:EGFP)* and angiogenesis inhibitors (SU5416 and sunitinib), it was elegantly shown that like in humans liver hyperproliferation is dependent on hypoxia and angiogenesis in a *myc*-induced zebrafish liver cancer model (80). In addition to the direct influence on tumor size, neovascularization could be important for metastasis, providing tumor cells entry to the vascular system.

90% of cancer patients die from metastases (81), so a treatment to inhibit the metastatic process would be a major breakthrough in cancer therapy. During the metastatic process, tumor cells switch their phenotype. Initially, often through epithelial–mesenchymal transition (EMT) tumor cells disseminate, migrate, and enter the blood circulation. After extravasation, they switch from an invasive to a proliferative phenotype. In melanoma, the invasive phenotype is associated with low and the proliferative/differentiated with high MITF levels and this phenotype switch is likely induced by the TME (58). A recent zebrafish study looked at the fate of melanoma cells during the metastatic process focusing on the regulation of cellular plasticity and differentiation by factors of the microenvironment (58). An initially unpigmented mesenchymal zebrafish melanoma cell line derived from *mitfa:BRAF^V600E^* melanomas regained pigmentation upon transplantation indicating differentiation. This was also associated with the upregulation of a differentiation signature of MITF target genes including EDNRB receptor. Using this cell line together with human melanoma cell lines, the authors identified endothelin EDN3, likely derived from keratinocytes, to induce phenotype switching leading to increased proliferation, melanin content, and differentiation. Inactivation of EDN3 and its converting enzyme ECE2 by CRISPR/Cas9 led to reduced tumor size and increased survival rates. Targeting TME factors like EDN3 promises to be a beneficial strategy to inhibit metastatic success.

The effect of wounding on cancer progression is an understudied but important topic, as surgery is a key cancer therapy and biopsies are the gold standard for diagnosis. Based on previous studies comparing the immune responses elicited by wounding and cancer formation, a recent study set out to investigate the direct effects of wounding on melanoma propagation in zebrafish (82). Indeed, the authors found that more than 40% of repeatedly wounded *kita:HRAS^G12V^* fish developed local tumors at the sites of wounding compared to unwounded fish. Wounding close to tumor sites was associated with an inflammatory response as macrophages and neutrophils were recruited not only to the wound but increasingly to adjacent tumor cells where they persisted for longer time periods. Wounding-induced proliferation of cancer cells could be blocked by morpholinos inhibiting myeloid cell development, suggesting that myeloid cells fuel the proliferation of cancer cells. In human cancer biopsies, the extent of ulceration, a negative prognostic marker, correlated with the number of infiltrating neutrophils but not macrophages. Based on this, improvements toward minimal invasive surgery and potential peri-operative anti-inflammatory treatment options should be considered.

These examples show that zebrafish has developed into a powerful model organism to study the TME. However, one caveat at early developmental stages best suited for *in vivo* microscopy investigations is that the adaptive immune system is not yet fully functional (83). Nevertheless, we have highlighted studies demonstrating the translational potential of zebrafish TME studies.

## Toward PDX in Zebrafish

Xenotransplantation of patient-derived tumor cells into zebrafish embryos and larvae for short-term cultivation, analysis, and compound screening is an appealing concept, as it promises to provide patient-specific insights and patient-tailored therapeutic strategies. Several groups have embarked on establishing protocols for xenotransplantation, initially using cultured tumor cell lines. In 2005, Lee et al. were the first to inject human melanoma cells into blastula stage zebrafish embryos (84). They maintained injected embryos at 31°C and tracked melanoma cells, which survived and divided in the fish for several days. At 5 days post fertilization (dpf) cells were observed in the head, trunk, and tail of injected fish.

Other groups have chosen 24 and 48 h post fertilization (hpf) for xenotransplantation and injected several hundred cells into the yolk, the Duct of Cuvier, the caudal vein, the pericardial cavity, the perivitelline space, and the ventricles of the brain (85–87). In addition, orthotopic xenografts have shown promising results (88). Injected tumor cell lines include glioma (89), HCC (90), lung cancer (91), pancreatic cancer (92), ovarian carcinomas (93), breast cancer (94), Ewing sarcoma (95–97), prostate cancer (98), retinoblastoma (99), and leukemia (100).

Due to the absence of an adaptive immune response until 4–6 weeks post fertilization (wpf), xenografted cells are not rejected at these early time points (83, 101). Typically, transplanted zebrafish are now maintained at 32.5–35°C relatively close to the physiological temperature of human cells, but still permitting normal zebrafish development (87). To visualize xenotransplanted cells for fluorescence microscopy, they are usually dye labeled, most often using CM-DiI (102).

Typical readouts allowing for quantification of the behavior of transplanted tumor cells in the fish host are proliferation, migration, and neovascularization. Proliferation of transplanted tumor cells can be investigated in a straightforward way by using available human-specific anti-ki67 antibodies (88, 103). Neovascularization can be visualized easily by performing xenotransplantation into transgenic zebrafish strains with fluorescently labeled vasculature (104, 105). Different tools have been applied for image-based quantification of migration, including ImageJ/Fiji open source and commercial software solutions, like Image-Pro Plus-based software MetaXpress (95, 96, 100, 106).

Teng et al. showed that the migratory/spreading behavior of transplanted cells in zebrafish correlated well with their metastatic potential *in vitro* (106). A preliminary experiment using short-term-cultured primary lung cancer cells confirmed that tumor cell spreading in zebrafish can be used as readout for metastatic potential (106). As about 90% of cancer patients die from metastatic spread of primary tumors, *in vivo* models complementary to the mouse model will be beneficial (107). Using a zebrafish melanoma xenograft model, it was demonstrated that poorly invasive cell populations coinvade with inherently invasive cells, thereby maintaining heterogeneity of melanoma cells (108).

As transplantation protocols of tumor cell lines become more robust (109), the field appears to be ready for real PDXz models, which were pioneered by Marques et al., who transplanted pancreas, stomach, and colon primary tumor cells into the yolk of 48 hpf zebrafish embryos (102). More recent reports using primary cultures of breast cancer cells from bone metastases and neuroendocrine tumor cells and spheroids obtained from papillary thyroid cancer fuel the hope for personalized medicine approaches using short-term PDXz (110–112). Especially the low number of cells used for transplantation into zebrafish might allow one to use tumor cells of low abundance, such as disseminated tumor cells. Nevertheless, it needs to be investigated, how well tumor heterogeneity is preserved in PDXzs and how the zebrafish environment changes gene expression and behavior of transplanted human tumor cells. The future will also tell how well actual primary cells engraft into zebrafish embryos/larvae and if there is need for “humanizing” zebrafish to be able to provide lacking growth factors. For some slowly growing primary tumor cells, the short experimental setup proposed for PDXzs might actually be disadvantageous. Here, several immunocompromised zebrafish strains like *rag2^E450fs^, jak3^P369fs^, prkdc^D3612fs^*, and *zap70^y442^*, which can be combined with optically clear mutant strains like casper, will help to overcome adaptive immune response and imaging problems associated with performing xenograft studies in juvenile zebrafish (21, 113–115).

## Strategies for Identifying Potential Therapeutic Compounds by Gezm or PDXz Drug Screens

Toxicology and toxicity studies using various fish models have a long tradition due to the ease of substance administration directly into the water and easy-to-recognize developmental malformations as readout (116). In 2000, a pioneering screen demonstrated that small molecule effects on organ development can be studied in whole zebrafish larvae in 96 well format (117). In the following 15 years, nearly 100 zebrafish screens were conducted with differing strategies, functional focus, and compound library size as reviewed by Rennekamp and Peterson (118). Generally, phenotype-based screens have higher success rates than target-based screens, which led to great interest in compound screening using zebrafish models related to human diseases (119).

Design and especially the readout is crucial for the success of a screen. In the following, we will present recent approaches relevant to the field of cancer, including screens based on developmental surrogate readouts, high-throughput screens using GEZMs and signal pathway-targeted screens.

### Screens Using Developmental Surrogate Markers

As zebrafish has been used in developmental biology for decades, the extensive knowledge can now be exploited for drug screens. During tumorigenesis, developmental programs are reactivated to escape anti-proliferative mechanisms like contact-inhibition, fate commitment, or apoptosis (72). Screens using developmental processes as readout can therefore be informative for oncology.

EMT is tightly connected to metastatic behavior of cancer cells and as metastasis is causing the majority of deaths related to cancer, therapeutic strategies blocking this process would be highly beneficial. Complex situations such as cells leaving their epithelial context are ideally modeled *in vivo*. Toward this goal, a transgenic zebrafish strain [*Tg(snai1b:GFP)*] was generated, which labels epithelial cells undergoing EMT to produce cells of the neural crest lineage (120). Applying this strain in a chemical compound screen revealed that TP-0903 is able to strongly inhibit EMT (120). Interestingly, TP-0903 is a multi-kinase inhibitor and subsequent testing of single target kinases was not able to generate the same effect. This emphasizes the tightly orchestrated activity of several kinases during EMT, which is likely true for many other biological processes. Eventually RNA sequencing and chemical rescue experiments showed that TP-0903 acts through stimulation of retinoic acid synthesis in this setting.

Another screen for potentially anti-metastatic compounds was carried out exploiting parallels between migrating posterior lateral line primordium (PLLp) cells in zebrafish and the behavior of invasive cancer cells (121, 122). Approximately 3,000 compounds were screened for their potential to affect PLLp migration in transgenic *Tg(cldnb:EGFP)* zebrafish, which express GFP in the PLLp and hereby offer a convenient readout (123). Approximately 5% of tested compounds had an effect without overt toxicity and among these was the Src inhibitor SU6656. The target of SU6656 could be rapidly validated using a CRISPR sgRNA targeting *src*. Finally, the authors showed that spreading of highly metastatic cells could be inhibited in a mouse orthotopic transplantation model, confirming that SU6656 has strong anti-metastatic activity in mammals as well.

In a leukemia-targeted screen, Ridges et al. reasoned that immature T cells might be a good surrogate for leukemic cells and thus used a transgenic strain [*Tg(lck:GFP)*], which demarcates immature T cells by GFP expression in developing zebrafish larvae (124). They screened more than 26,000 compounds on larvae in 96 well format using GFP expression in the thymic region as readout. Among the compound hits, they found lenaldekar to selectively eliminate immature T cells in larvae and also to prevent cMYC-induced T cell acute lymphoblastic leukemia (T-ALL) in adult zebrafish. Furthermore, lenaldekar was effectively inhibiting human leukemic xenograft growth in mice. This demonstrates how lead compounds can be identified in zebrafish screens.

### Screens On Genetic Cancer Models

An increasing number of specific zebrafish models for solid tumors as well as leukemias have been successfully employed in small compound screens. In these phenotype-based screens, rescue of disease-related malformations is often used as readout. With this holistic approach, no prior knowledge about pathological mechanisms is required for random library screening. In addition, a whole organism screen selects for compounds with low toxicity.

One recent screen was performed on a HCC model driven by liver-specific expression of β-catenin and histologically highly similar to the human disease (61). Using this model, the authors revealed that constitutive WNT/β-catenin signaling forces proliferation and consequently measurable increase in liver size *via* JNK signaling. In a high-throughput drug repurposing screen using excessive liver growth as readout, JNK inhibitors as well as unexpectedly specific anti-depressants were identified as potent inhibitors of liver growth. This suggests JNK inhibitors and specific anti-depressants as new therapeutic strategies for β-catenin-induced liver cancers.

Repurposing studies of already approved drugs promise a fast track into the clinics, as tolerance and side-effects in humans have already been investigated for these compounds. Testing for synergy of approved drugs on zebrafish cancer models promises alternative treatment options for cancer entities where monotherapy fails.

Synergistic effects were detected in a *Ras*^G12V^-driven melanoma model, which was elegantly analyzed on a standard plate reader measuring increased melanophore density. In a focused compound screen, combinations of the MAPK inhibitor PD184352 and the PI3K/mTOR inhibitors BEZ235 or rapamycin efficiently inhibited melanoma growth at low concentrations, where single compound treatment was not effective anymore (59).

Combined suppression of MAPK and PI3K/mTOR pathway also acted synergistically in a rhabdomyosarcoma zebrafish model (125). From nearly 3,000 tested drugs, the chymotrypsin-like serine protease inhibitor TPKC was found to inhibit S6k1, a downstream target of mTOR. Here, tumor growth could be suppressed with additional treatment with the MEK inhibitor PD96059.

Furthermore, He et al. highlighted in the already mentioned neuroblastoma model driven by aberrant MYCN expression and loss of NF1 that monodrug treatment is unlikely to achieve satisfying therapeutic effects. Their fish model was well suited to determine balanced and effective compound combinations. Applying isobologram analysis they worked out the best synergistic concentrations for MEK inhibitor trametinib and isotretinoin (46).

### Signaling Pathway Activity-Based Screens

In a more targeted approach, comparative strategies and available transgenic signaling pathway reporter strains can be used to identify compounds able to inhibit or augment a specific signaling pathway. As many signaling pathways play crucial roles in tumorigenesis, this is directly relevant for cancer management.

The Notch signaling pathway controls cell fates and orientation during development by direct cell–cell contact and is often deregulated during cancer pathogenesis.

A screen comparing the effects on larval development of more than 200 compounds to the standard Notch inhibitor DAPT identified 2 novel Notch inhibitory compounds. These small compounds also successfully reduced proliferation of human oral cancer cell lines *in vitro* (126).

Likewise, the Hedgehog (Hh) signaling pathway is essential during development and is also connected to several malignancies, including medulloblastoma and basal cell carcinoma (BCC). A Smoothened (Smo) antagonist is approved for the treatment of advanced BCC, but additional Hh pathway inhibitors downstream of Smo could define new strategies for the treatment of other Hh-dependent malignancies. Testing 30,000 compounds for their effects on zebrafish patterning, Williams and colleagues discovered eggmanone, a small molecule, mimicking the Hh null phenotype in zebrafish embryos (127). They could show that eggmanone is an inhibitor for a phosphodiesterase 4 isoform (PDE4D3). Strikingly, PDE4 has also been implicated as a driver of CNS tumors like medulloblastoma (128). Identifying PDE4 as a potent target to inhibit Hh-signaling opens up new possibilities in Hh-dependent cancer therapy.

In another elegant compound screen, the FGF signaling reporter strain *Tg(dusp6:EGFP)^*pt6*^* was used to identify compounds altering FGF signaling. (*E*)-2-benzylidene-3-(cyclohexylamino)-2,3-dihydro-1*H*-inden-1-one (BCI) augmented EGFP expression in this reporter strain. Mechanistically, BCI was found to block Dusp6 activity and enhance FGF target gene expression. Furthermore, a temporal role of Dusp6 during heart formation was discovered by treating zebrafish embryos with BCI at several developmental stages (129). Dusp6 is a phosphatase involved in the MAPK pathway, a pathway also often deregulated during cancer pathogenesis. Therefore, regulators of this target are of clinical relevance in oncology and BCI is indeed used in leukemia treatment.

### Remaining Questions For Zebrafish Model-Based Compound Screening

Many small compound screens have been performed, but our understanding of pharmacokinetic processes in zebrafish is still limited. Pilot studies address the important question if zebrafish larvae metabolize drugs in a way comparable to humans. Proteomics and transcriptomics analysis show a high degree of conservation of metabolic enzymes between human and zebrafish larvae, including key metabolic cytochrome P450 (CYP) genes (130, 131). In addition, liver, kidney, and blood–brain barriers are present in zebrafish larvae (119). Investigating testosterone metabolism using zebrafish larvae and liver microsomes from adult zebrafish (ZLM) indicated that more metabolic enzymes are present in adult fish. Comparing adult fish to human, the main testosterone metabolite was identical, but differences in minor metabolites were detected (132). Another study measured the pharmacokinetics of paracetamol metabolites in zebrafish larvae at 3 dpf. Paracetamol clearance rates scaled reasonably well with higher vertebrates and were similar to young humans (133). Clearly, additional investigations are needed to acquire a more complete understanding of pharmacokinetic processes in zebrafish larvae. Nevertheless, most importantly for the use of zebrafish compound screens, pharmacological effects were so far found to be well conserved between zebrafish and mammals. For example, 22 out of 23 known cardiotoxic drugs also exhibited repolarization-related toxicity when tested in zebrafish embryos (119, 134). *Vice versa* 8 out of 10 compounds first identified in zebrafish also produced the expected effect in rodents, suggesting a good translatability (119).

## Shortcomings and Challenges of the Zebrafish Model

In every model, some aspects of the process of interest are not well conserved and awareness of such shortcomings of the respective model is important (Table 2).

**Table 2:** Benefits and shortcomings of the zebrafish in cancer modeling.

	Benefits	Shortcomings
Embryonic development	Largely conserved development	Absent organs: breast, prostate, lung. Organ structure not as complex
	External embryonic development	
	Fast development: major organs formed within 48 hpf, cancer studies in larvae feasible	
Physiology	Optical transparancy of larvae facilitates imaging and high-throuput screening	Patient-derived xenograft (PDX): conservation of molecular interactions between transplanted human cells and zebrafish cells unclear
	Conserved signaling pathways	Zebrafish physiological temperature: 28/29°C, for PDX increased to 34–35°C; influence on tumor cell behavior unclear
		Studying of some drug side-effects such as fever compromised
Genetics	>80% of human disease-related genes present	Teleost-specific whole genome duplication: gene duplications can complicate studies
	Easy genetic manipulation: many transgenic reporter and driver lines for cancer models available	
	Transient manipulation of cancer pathways through injection into one-cell stage larvae possible	
Immune system	Underdeveloped adaptive immune system in larvae: no rejection of xenografts	Underdeveloped adaptive immune system in larvae: obstacle for studying fully functional TME
Tumor anatomy/histology	Many tumor models show comparable histology to human cancers	Genetic tumor models for breast, prostate, or lung cancer not possible
Handling and husbandry	Abundant larvae for drug screens (up to ~200 eggs/couple and week)	
	Easy and cost-effective drug screens	

Physiological differences with implications for cancer modeling exist between human and zebrafish. Organs like lung, breast, and prostate are missing in zebrafish, which hampers the generation of genetic cancer models for these tumor entities in zebrafish. Furthermore, orthotopic transplantation of tumor cells from these organs is not possible in zebrafish.

In addition, there are genetic differences. A teleost-specific whole genome duplication event resulted in the presence of ~26,000 protein-coding genes in zebrafish (~20,000 in human) and thus more than one ortholog for some human genes exists (6). This gene duplication potentially leads to redundancy or specialization in gene function and can complicate loss-of-function studies of tumor-suppressor genes. On the contrary, orthologs of some cancer-related genes like oncostatin M (OSM) or leukemia inhibitory factor (*LIF*) have not yet been identified in zebrafish. As corresponding receptors are encoded in the zebrafish genome, it is likely that orthologs for *LIF and OSM* with sequence divergence but similar protein function will be discovered in the future, but other genes like *CDKN2A* might actually be missing (86).

The zebrafish genome size (1.4 Gb) is around half of the human genome size and differences are also found in non-coding regions. Type 1 (retrotransposable elements) cover 44% of the human sequence, but only 11% of the zebrafish genome. In contrast, type II (DNA transposable elements) cover 3.2% of the human but 39% of the zebrafish genome (6). It is currently unclear if this difference in types of transposable elements found in human and zebrafish genomes has implications for cancer modeling in zebrafish.

Ontogeny and function of innate and adaptive immune cells is highly conserved between human and zebrafish. However, a functional adaptive immune system is not present in zebrafish larvae within the first 4 weeks after fertilization (83, 101). Thus, the role of adaptive immune cells in tumor initiation and progression cannot be studied in zebrafish cancer models at these early stages.

Nevertheless, the absence of an adaptive immune response allows xenografts to be carried out without immunosuppression in zebrafish larvae.

A challenge for establishing PDXz models is the slightly cooler temperature in zebrafish larvae (32.5–35°C instead of 37°C), which might affect the behavior of transplanted human tumor cells. Furthermore, some zebrafish growth factors might not be conserved enough to support growth of specific tumor cells. *Vice versa*, it is known that human growth factors do not support zebrafish hematopoiesis *in vitro* (135). Therefore, similar to mouse xenograft models, humanizing zebrafish might be necessary in the future to improve xenotransplantation success rates.

## Quo natas, Danio?

As outlined earlier, several novel and improved genetically engineered zebrafish cancer models have been generated over the last couple of years. Many of them have provided new mechanistic insights into tumorigenesis.

We have highlighted elegant screening strategies using zebrafish cancer models, but also developmental process- and signaling pathway-targeted approaches, which have identified chemical inhibitors and their synergistic effects, when applied in combination, of different aspects of tumorigenesis. Phenotype-based compound screening in zebrafish is also ideal for recent polypharmacology strategies to discover single drugs with effects on multiple targets. In the future, automation of the entire small compound screening process including zebrafish handling, image acquisition and image analysis will allow for higher throughput screens and several solutions are already available (136–139).

In addition to small compound screens, first automated injection examples promise that rapid testing of biologics and their delivery vehicles is also feasible in zebrafish (140).

Taken together, zebrafish models have proven to be valuable for cancer research offering unique opportunities, which are complementing mouse and human systems. Current areas of great interest in cancer research including the TME, cancer immunotherapy, epigenetics, and precision medicine will become important topics in zebrafish cancer modeling.

Live imaging together with genetic manipulation of tumor cells and their microenvironment in zebrafish will yield a better understanding of the contribution of each cell type to tumor progression. The innate immune system is already being investigated in a tumor context at larval stages. Applying fish strains, still transparent at juvenile and adult stages, will also allow for observation of adaptive immune cells by live microscopy in GEZMs. By such means, zebrafish cancer models will likely provide novel TME-targeted therapeutic strategies including immunotherapies.

While the cancer field for a long-time focused on how genetic changes lead to tumor formation, the significance of epigenetic control over gene regulation is now being recognized. Notably, pediatric cancers contain only a limited number of mutations, suggesting epigenetic aberrations as important tumor drivers. Epigenetic marks as well as the DNA methylation and histone modification machinery are well conserved in zebrafish, promising that zebrafish cancer models will become important tools to dissect the relevance of epigenetic changes in cancer cells *in vivo* (141).

Finally, with CRISPR/Cas9 genome editing possibilities, personalized genetic zebrafish cancer models harboring patient-specific mutations will be generated. The next couple of years will also reveal the potential of PDXz. Eventually, personal cancer fishes, encompassing GEZMs and PDXzs might be used to characterize individual malignancies and to test compounds in personalized cancer medicine approaches in the not too distant future.

## Author Contributions

SK, CS, SP, and MD all contributed to writing of this review.

## Conflict of Interest Statement

The authors declare that the research was conducted in the absence of any commercial or financial relationships that could be construed as a potential conflict of interest.
